# BALB/c mice infected with DENV-2 strain 66985 by the intravenous route display injury in the central nervous system

**DOI:** 10.1038/s41598-018-28137-y

**Published:** 2018-06-27

**Authors:** Natália G. Salomão, Kíssila Rabelo, Tiago F. Póvoa, Ada M. B. Alves, Simone M. da Costa, Antônio J. S. Gonçalves, Juliana F. Amorim, Adriana S. Azevedo, Priscilla C. G. Nunes, Carlos A. Basílio-de-Oliveira, Rodrigo P. Basílio-de-Oliveira, Luiz H. M. Geraldo, Celina G. Fonseca, Flávia R. S. Lima, Ronaldo Mohana-Borges, Emiliana M. Silva, Flávia B. dos Santos, Edson R. A. Oliveira, Marciano V. Paes

**Affiliations:** 1 Laboratório Interdisciplinar de Pesquisas Médicas, Instituto Oswaldo Cruz, Fundação Oswaldo Cruz, Rio de Janeiro, Brazil; 2 Laboratório de Ultraestrutura e Biologia Tecidual, Universidade do Estado do Rio de Janeiro, Rio de Janeiro, Brazil; 3 Instituto de Criminalística, Tocantins, Brazil; 4 Laboratório de Biotecnologia e Fisiologia de Infecções Virais, Instituto Oswaldo Cruz, Fundação Oswaldo Cruz, Rio de Janeiro, Brazil; 5 Laboratório de Tecnologia Virológica, Instituto de Tecnologia em Imunobiológicos, Fundacão Oswaldo Cruz, Rio de Janeiro, Brazil; 6 Laboratório de Imunologia Viral, Instituto Oswaldo Cruz, Fundação Oswaldo Cruz, Rio de Janeiro, Brazil; 7 Anatomia Patológica, Hospital Gaffrée Guinle, Universidade Federal do Estado do Rio de Janeiro, Rio de Janeiro, Brazil; 8 Laboratório de Biologia das Células Gliais, Instituto de Ciências Biomédicas, Universidade Federal do Rio de Janeiro, Rio de Janeiro, Brazil; 9 Laboratório de Genômica Estrutural, Instituto de Biofísica Carlos Chagas Filho, Universidade Federal do Rio de Janeiro, Rio de Janeiro, Brazil; 10 Laboratório de Imunologia Viral, Instituto Oswaldo Cruz, Fundação Oswaldo Cruz, Rio de Janeiro, Brazil; 11 Laboratório de Modelagem Molecular, Instituto de Química Orgânica, Universidade Federal do Rio de Janeiro, Rio de Janeiro, Brazil

## Abstract

Dengue is a mild flu-like arboviral illness caused by dengue virus (DENV) that occurs in tropical and subtropical countries. An increasing number of reports have been indicating that dengue is also associated to neurological manifestations, however, little is known regarding the neuropathogenesis of the disease. Here, using BALB/c mice intravenously infected with DENV-2 strain 66985, we demonstrated that the virus is capable of invading and damaging the host’s central nervous system (CNS). Brain and cerebellum of infected animals revealed histological alterations such as the presence of inflammatory infiltrates, thickening of pia matter and disorganization of white matter. Additionally, it was also seen that infection lead to altered morphology of neuroglial cells and apoptotic cell death. Such observations highlighted possible alterations that DENV may promote in the host’s CNS during a natural infection, hence, helping us to better understand the neuropathological component of the disease.

## Introduction

Dengue is a mosquito-borne disease that represents a major health problem especially in tropical and subtropical regions worldwide. The disease is caused by dengue virus (DENV), which comprises four antigenically different serotypes (DENV-1 to DENV-4) belonging to the *Flaviviridae* family. Dengue burden has been expanding since 1960s as it grew side by side with the world’s population. Nowadays, around 390 million people are infected every year, of which about 25% are of clinical relevance^1^. Symptoms of dengue are usually similar to the regular flu, however, a small fraction of cases may evolve to a severe hemorrhagic form that is eventually responsible for about 20,000 deaths in an annual basis^2,3^.

An intriguing fact that has drawn attention in dengue is the involvement of the host’s central nervous system (CNS) in the course of infection. CNS-related symptoms of dengue were first reported as an acute encephalopathy in 1976^4^ and, classically, these manifestations have been treated as rare phenomena in humans^5,6^. Back in 1998, Ramos and coworkers suggested that DENV neurotropism was based on opportunism^7^. However, a growing number of reports showing the presence of the virus in the host’s CNS^8–12^ supported the idea that the neurotropic property would be an intrinsic characteristic of the virus. After being vastly reported in 25 countries spread across different continents^13–22^, neurological signs in dengue were grouped into 3 sub-classifications: (i) encephalopathy; (ii) neuromuscular complications; and (iii) neuro-ophthalmic involvements. The incidence rate of such symptoms varied from 0.5 to 20% among patients admitted to hospitals^23^. Additionally, neurological signs of subjective nature and of difficult characterization, such as restlessness, irritability, dizziness, drowsiness and stupor, were also associated to the disease^24^. Considering the increased frequency of CNS-related symptoms in patients with dengue, neurological manifestations were officially recognized by the World Health Organization (WHO) and listed as part of the differential diagnosis for severe dengue in 2009^25^. Despite this official recognizance, information regarding the neuropathological basis of the disease is still scarce and demanding of research.

In an attempt to describe the impact of DENV infection in the host’s CNS, BALB/c mice were infected with patient-isolated DENV-2 by the intravenous (i.v.) route. Despite the absence of apparent symptoms, mice responded to the infection with antigen presenting cell (APC) induction and TCD8 cell activation. Histological analyses of the infected animals’ brain and cerebellum revealed pathological alterations such as presence of inflammatory infiltrates, thickening of pia matter and disorganization of white matter in the brain; and altered Purkinje neurons, hemorrhage and demyelination in the cerebellum. Analysis of microglia (IBA-1^+^) and astrocytes (GFAP^+^) in the brain showed that these subpopulations were morphologically altered in infected mice. Electron microscopy analyses of brain and cerebellum from DENV-infected mice revealed cellular impairments that suggested apoptotic induction. We also found that DENV was able to reach the host’s CNS upon i.v. inoculation, as detected by DENV-NS3 staining, supporting the natural neurotropic behavior of the virus and suggesting that the observed effects were due to direct viral infection in the CNS. Such findings highlighted possible brain alterations that could yield CNS-related symptoms in dengue and helped us to better understand the neuropathological component of the disease.

## Results

### DENV infection in BALB/c mice induces TCD8-mediated cellular immunity

In the experiments considered here, BALB/c mice were infected with patient-isolated DENV-2 by the intravenous (i.v.) route. It was previously demonstrated that although the infection is not capable of producing symptoms, it can induce hepatic injury in the subjects and virus can be detected in different cell types such as hepatocytes, Kupffer cells and endothelium^26^. Given the influence that the cellular immunity may have towards the neurological involvement in dengue^17,19,27^, our initial step here was to address the immunological status of these mice upon the i.v. infection.

**Figure 1:**
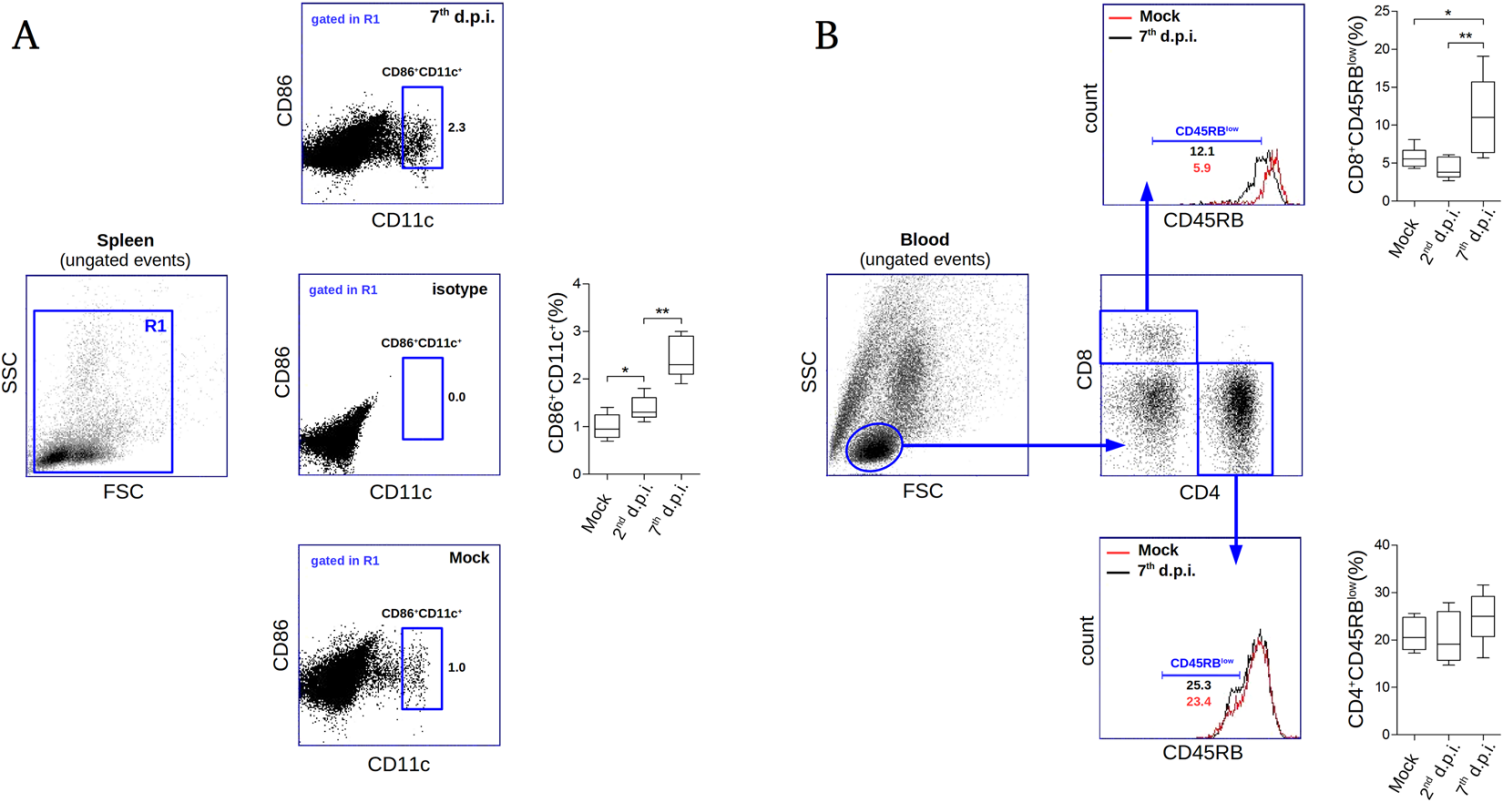
Flow cytometry analysis of spleen and blood samples from DENV-infected mice. Groups of BALB/c mice (n = 6 each), mock, 2^nd^ and 7^th^ days post infection (d.p.i.) were considered for flow cytometry evaluation using individual spleen and blood samples. **(A)** Isolated splenocytes were stained with anti-CD86-FITC and anti-CD11c-PE and read into a BD Accuri C6 flow cytometer. Only events clustered into the R1 region in the forward and side light scattering representation (FSC × SSC) were took into account for this analysis (left). Based on the isotype control, a CD86^+^ CD11c^+^ region (considered as the antigen presenting cell subset) was defined (center). Representative flow cytometry dotplots regarding mock and infected samples, as well as the quantitative analysis between groups are shown (bottom, top, and right, respectively). **(B)** Flow cytometry analysis showing leukocytes isolated from blood stained with anti-CD8-PerCP, anti-CD4-PE and anti-CD45RB-FITC. A region of lymphocytes in the FSC × SSC representation was defined (left) for subsequent specification of CD4^+^ and CD8^+^ regions (center). Expression of CD45RB was investigated within the CD4^+^ and CD8^+^ regions considering non-infected and infected groups (bottom and top, respectively). Statistical analyses are shown for each evaluation (top right and bottom right). Statistical differences were evaluated using Mann-Whitney test (*p < 0.05; **p < 0.01).

Spleen and blood samples of infected animals were evaluated by flow cytometry two and seven days post infection (d.p.i.). When analyzing the spleen of infected mice, we observed relevant increments in the percentages of CD86^+^ CD11c^+^ cells (which we considered as the antigen presenting cell subset - APC) on the 2^nd^ and on the 7^th^ d.p.i., in comparison to controls. Of note, on the 7^th^ d.p.i. the percentage of CD86^+^ CD11c^+^ cells reached approximately 2 times higher than the observed in the mock-infected group (Fig. 1A). This finding suggested that the i.v. infection induced activation and migration of APCs to the lymphoid organ in order to trigger specific immunity. Under this line of thinking, we next checked the levels of activated lymphocytes present in the circulation of the infected mice. Flow cytometry data revealed that on the 7^th^ d.p.i. the percentage of CD8^+^ CD45RB^low^ (considered as the activated TCD8 cells) consistently increased when compared to mock or to the 2^nd^ d.p.i. groups (Fig. 1B). No statistically relevant variations were seen in the percentages of circulating CD4^+^ CD45RB^low^ cells considering all the analyzed groups. These data indicated that, upon infection, the animals were able to trigger specific immunity to DENV-2 with the activation of TCD8 lymphocytes.

### BALB/c mice present damage in the CNS after DENV infection by the i.v route

In the previous evaluation, the occurrence of activated lymphocytes in the circulation of infected mice draw our attention for a possible targeting of T-cell migration to host’s tissues. To address the involvement of infection and a possible impact of the cellular immunity in the CNS we proceeded with histological studies considering the brain and the cerebellum tissues. Four major areas were took into account for investigation: (i) the cerebral cortex, which comprises the sensory and motor areas of the brain; (ii) the hippocampus, which consists of an internal area of the brain related to memory; (iii) the cerebral white matter, a region of myelinated nerve fibers that is critical for the axonal signaling and flux; and (iv) the cerebellum, a structure that plays a crucial role in motor control^28^.

**Figure 2:**
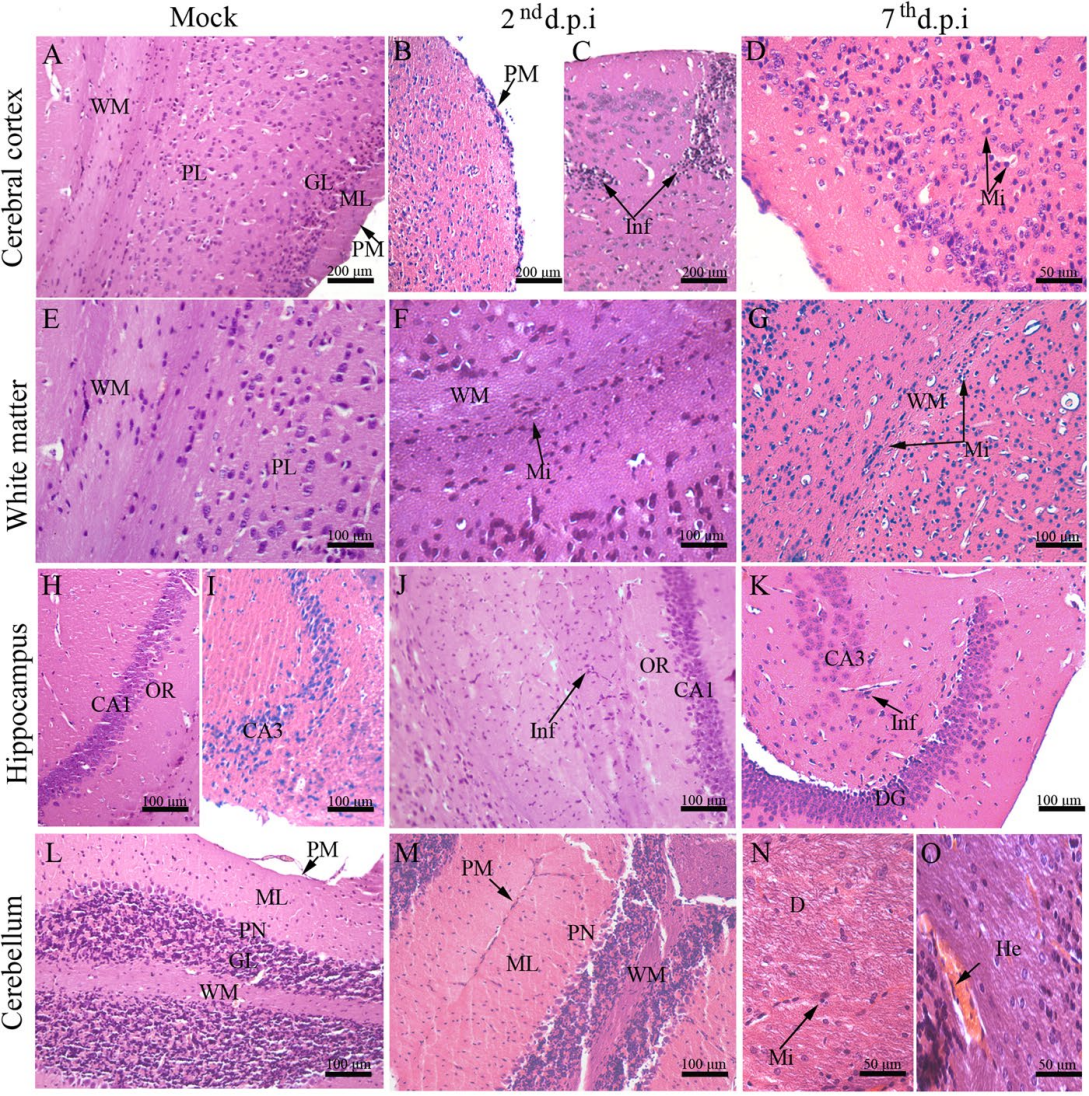
Histopathological aspects of the brain and cerebellum tissues of DENV-infected mice. (**A)** Cerebral cortex of a mock-inoculated mouse exhibiting normal aspects; **(B)** Mouse infected with DENV-2 showing pia mater with inflammatory infiltrate; **(C)** focal perivascular inflammatory infiltrate on the 2^nd^ d.p.i and **(D)** diffuse inflammatory infiltrate on the 7^th^ d.p.i. **(E)** Normal white matter from a mock-inoculated mouse. **(F)** White matter committed with inflammatory infiltrate on the 2^nd^ d.p.i and **(G)** on the 7^th^ d.p.i. **(H)** CA1 and **(I)** CA3 hippocampal regions from a mock-inoculated mouse. **(J)** Microglial cell infiltrate in CA1 on the 2^nd^ d.p.i and **(K)** in CA3 on the 7^th^ d.p.i. **(L)** Cerebellum region with normal aspects extracted from a control mouse. **(M)** Degenerated Purkinje neuronal layer on the 2^nd^ d.p.i. **(N)** Demyelination with microglial cell infiltrate and **(O)** hemorrhage on the 7^th^ d.p.i. CA1 - *Cornu ammonis* 1 region; CA3 - *Cornu ammonis* 3 region; D - demyelination; DG - dentate gyrus; GL - granular layer; H - hippocampus; He - hemorrhage; ML - molecular layer; OR - orien; PL - pyramidal layer; PM - pia mater; Inf - Inflammatory infiltrate; PN - Purkinje neuron; Mi - Microglia; WM - white matter; d.p.i. - days post infection.

Considering the samples from infected mice, histopathological analyses showed damage in the brain and cerebellum tissues. On the 2^nd^ d.p.i., the cerebral cortex showed thickening of the pia mater with an increased cellularity (Fig. 2B) and focal perivascular inflammatory infiltrate consisting mainly of lymphocytes and glial cells (Fig. 2C). On the 7^th^ d.p.i., the inflammatory infiltrates were more diffuse by the parenchyma with predominance of microglial cells (Fig. 2D). As expected, brain of non-infected mice showed pia mater, molecular layer, granular layer, pyramidal neurons layer and white matter with regular structures (Fig. 2A). When analyzing the white matter from infected mice we found that this region was structurally disorganized. On the 2^nd^ d.p.i. this area was marked by microglial cell infiltrates within the parenchyma (Fig. 2F), while later on the 7^th^ d.p.i. the cell infiltrates were more characterized around blood vessels (Fig. 2G). Regarding the hippocampus area, while non-infected mice exhibited regular structures (Fig. 2H,I), infected animals showed presence of neuroglial cells infiltrates within this region. In this case, on the 2^nd^ d.p.i. infiltrates were seen in the CA1 (Cornu Ammonis 1) region (Fig. 2J), while on the 7^th^ d.p.i. this manifestation occurred mainly in the CA3 (Cornu Ammonis 3) (Fig. 2K).

Analyses of the cerebellum tissue collected from infected animals revealed inflammatory infiltrates in the pia mater. Purkinje neurons were also characterized by altered morphology on the 2^nd^ d.p.i. (Fig. 2M). On the 7^th^ d.p.i., it was observed an extensive area of demyelination with presence of microglial cells in the white matter (Fig. 2N) and circulatory damage such as hemorrhage in the cerebellar parenchyma (Fig. 2O). Control mice showed cerebellum structures with regular aspects (Fig. 2L).

**Figure 3:**
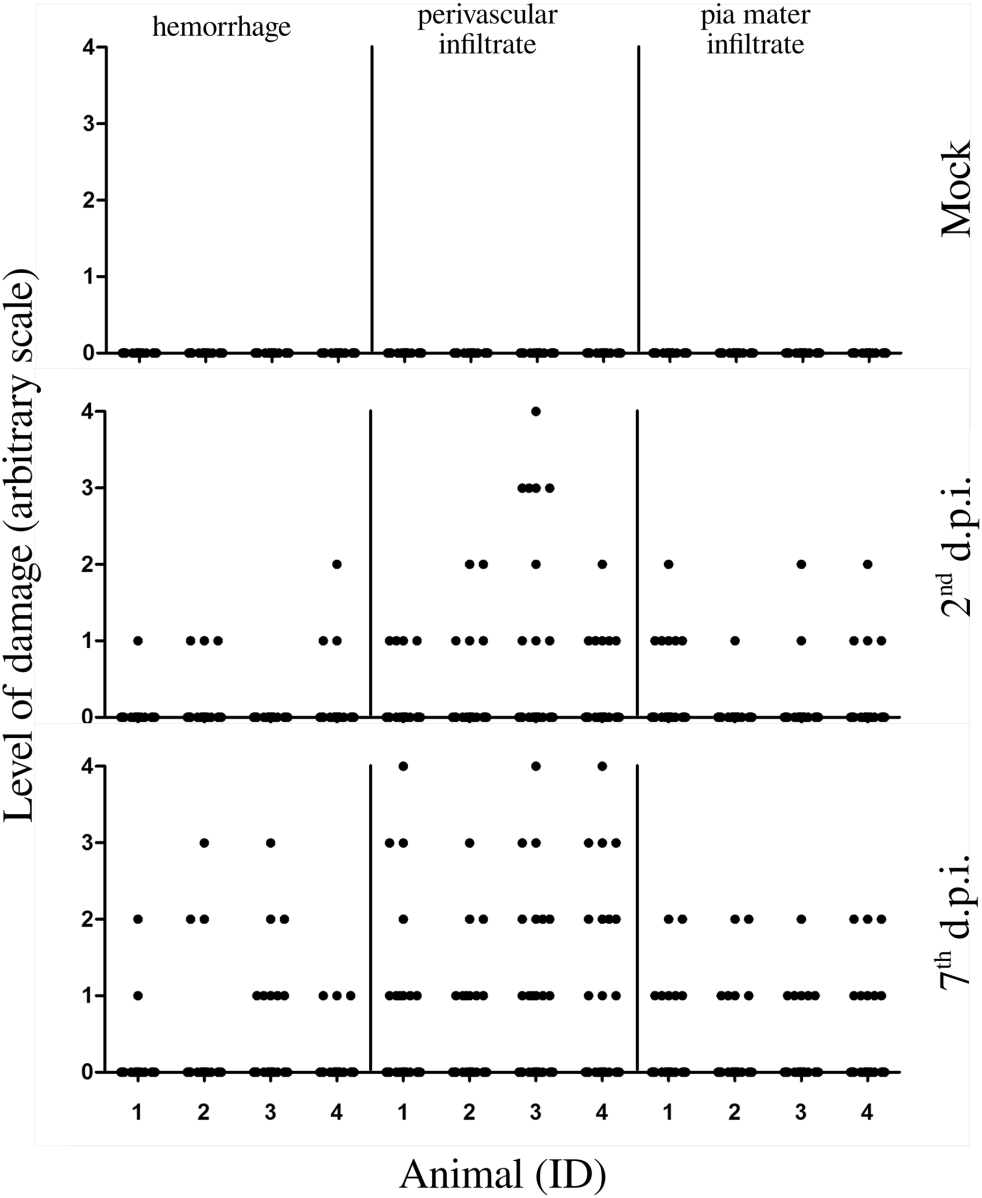
Quantification and qualification of damage in the brain of infected mice. Groups of BALB/c mice (n = 4 each), mock, 2^nd^ and 7^th^ d.p.i. were considered for quantification and qualification of damage in their brains. Three types of tissue damage were taken into account: (i) hemorrhage; (ii) perivascular infiltrate; and (iii) pia mater infiltrate. 30 fields of HE-stained histological cuts at magnification of 400× were evaluated according to an arbitrary scale as follows: 0 - absent; 1 - light and focal; 2 - light; 3 - moderate; and 4 - diffuse. Raw data is available in the Supplementary Information, Table 1. d.p.i. - days post infection; ID - identification number of animal.

In order to identify the extent of brain damage caused by DENV-2 strain 66985, three histopathological parameters were quantified: (i) hemorrhage; (ii) perivascular infiltrate; and (iii) pia mater infiltrate. Although the infection could lead to brain hemorrhage, this morphological change was mostly focal and limited to small areas. In average, on the 2^nd^ d.p.i. there were only 2 positive fields out of 30, while on the 7^th^ d.p.i. this damage was found in a more spread fashion (in average 4 positive fields out of 30) but still rarely observed (Fig. 3 left). Perivascular and pia mater infiltrates behaved similarly, however at different magnitudes, being more evident on the 7^th^ d.p.i., when compared to the 2^nd^ d.p.i. In average, considering perivascular infiltrates, on the 2^nd^ d.p.i. there were approximately 6 positive fields out of 30, while on the 7^th^ d.p.i. this margin was increased up to 11 positive fields out of 30 (Fig. 3 center). Infiltrates in the pia mater were averaged as 4 positive fields out of 30 on the 2^nd^ d.p.i. and 7 positive fields out of 30 towards the 7^th^ d.p.i. (Fig. 3 right). No histological alterations were observed in the controls.

### Infected animals reveal morphological alterations of microglial cells and astrocytes

After characterizing tissue alterations in the brain and cerebellum of DENV-infected animals, we considered investigating the brain cellular components responsible for tissue homeostasis. For this, we analyzed in more detail the neuroglial cells, in particular microglial cells and astrocytes, since they are known to play critical roles in maintaining CNS’ homeostasis, supporting and protecting neurons from injury^29,30^.

**Figure 4:**
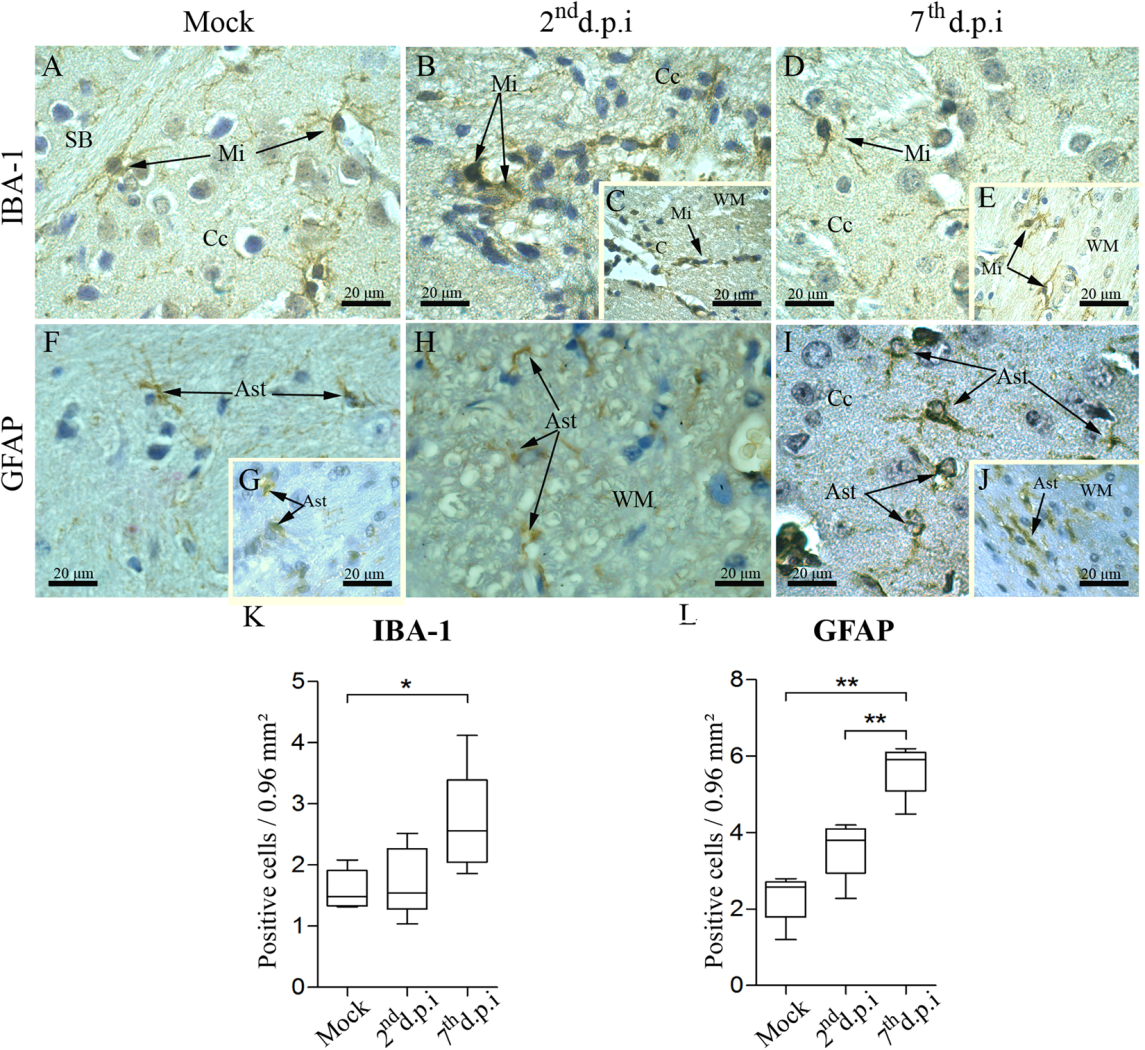
Aspects of microglia and astrocytes in brain tissues of infected mice. Detection of microglia (IBA-1^+^ cells) in samples of the cerebral cortex and white matter from **(A)** non-infected, **(B/C)** 2^nd^ d.p.i and **(D/E)** 7^th^ d.p.i. **(F/G)** Detection of astrocytes (GFAP^+^ cells) in the cerebral cortex and white mater in samples from control animals. **(H)** Staining of astrocytes in the white matter of samples from infected mice on the 2^nd^ d.p.i. **(I/J)** Staining of astrocytes in the cortex and in the white matter on the 7^th^ d.p.i. Quantification of **(K)** IBA-1^+^ and **(L)** GFAP^+^ cells. Ast - astrocyte; C - capillary; Cc - cerebral cortex; Mi - microglia; WM - white matter; d.p.i. - days post infection. Statistical differences were evaluated using Mann-Whitney test (*p < 0.05; **p < 0.01).

In samples collected from non-infected mice, microglial cells (IBA-1^+^ cells) were detected in the cortex and white matter. This cell population was found exhibiting scarce cytoplasm, long/thin extensions (Fig. 4A) and assuming a ramified morphology, which is known to be typical of their surveillant and homeostatic state. In the samples from infected animals, these cells were characterized by ameboid morphology with retracted extensions and increased cytoplasm located in the cortex (Fig. 4B) as well as in the white matter close to the capillaries (Fig. 4C). This morphological characterization, which is typical from their activated state, was observed mainly on the 2^nd^ d.p.i. Based on morphology, later at the 7^th^ d.p.i. these cells seemed to return to their regular activity/processes (as seen by their ramified appearance) both in the cortex (Fig. 4D) and in the white matter (Fig. 4E). Astrocytes (GFAP^+^ cells) in non-infected mice showed thin appearance in the cortex (Fig. 4F) and in the white matter (Fig. 4G). In the samples from infected mice, GFAP^+^ cells were found under a degenerative process with atrophy of their cytoplasmic extensions on the 2^nd^ d.p.i. (Fig. 4H). On the 7^th^ d.p.i., astrocytes assumed large and thick shapes with thicker cytoplasmic extensions either in the cerebral cortex or in the white matter (Fig. 4I,J).

In order to identify whether the CNS was committed by reactive microgliosis or astrogliosis, we proceeded with the quantification of both IBA-1^+^ and GFAP^+^ cells within the cerebral cortex and white matter. As shown in the Fig. 4 panels k and l, both cell types statistically increased in number on the 7^th^ d.p.i., when compared to controls. This finding confirmed the occurrence of reactive microgliosis and astrogliosis upon infection with DENV by the i.v. route.

### Ultrastructural aspects of brain and cerebellum from DENV-infected mice

**Figure 5:**
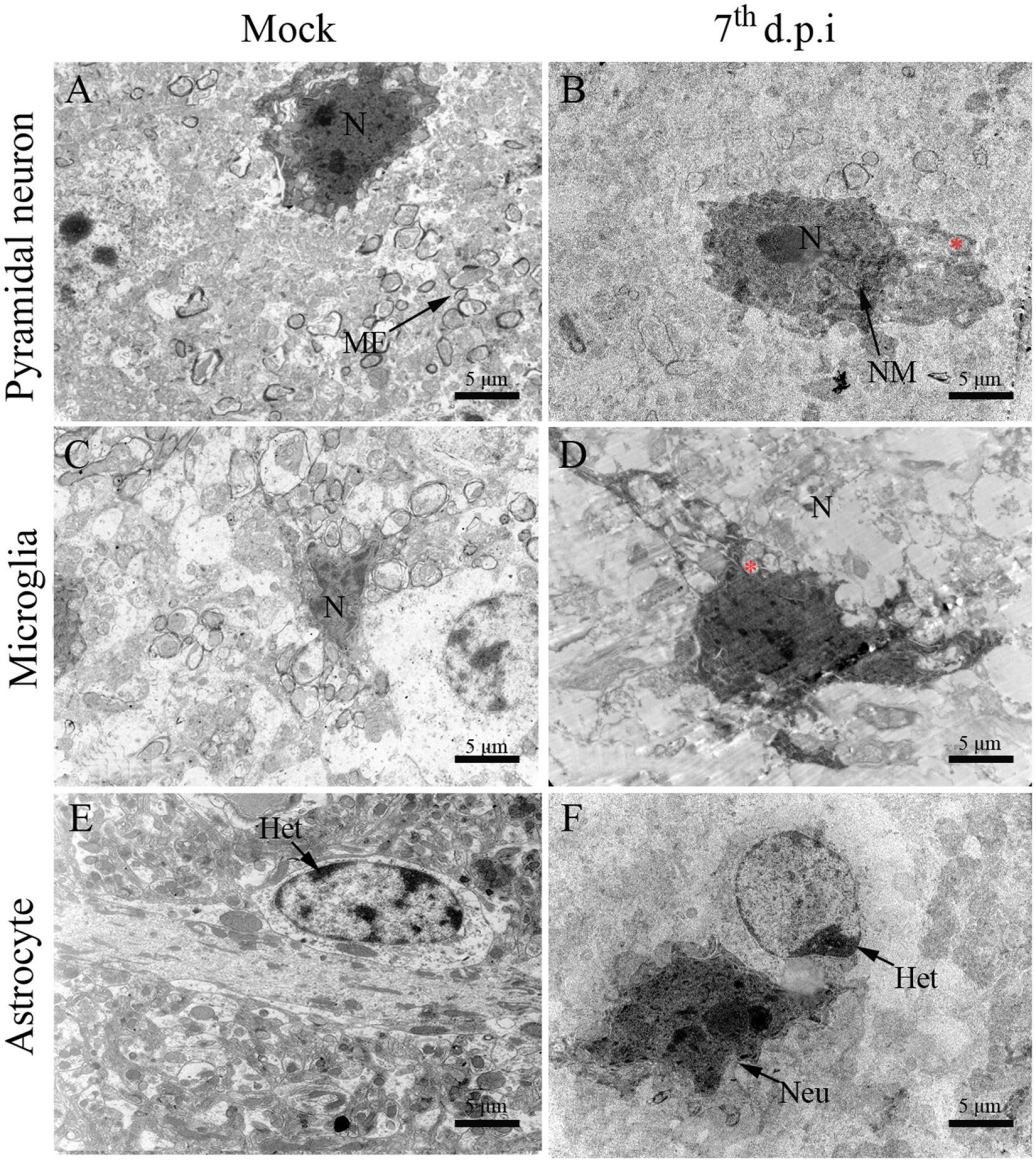
Ultrastructural aspects of the brain tissue of BALB/c mice infected with DENV-2. (**A)** Pyramidal neurons, **(C)** microglial cells and **(D)** astrocyte of non-infected mice showing regular aspects. **(B)** Pyramidal neuron exhibiting increased nucleus, swollen mitochondria and irregular nuclear membrane. **(D)** Microglial cell with increased nucleus and swollen mitochondria. **(F)** Astrocyte with heterochromatin deposition. Samples from infected mice were considered on the 7^th^ d.p.i. Het - heterochromatin; (Red asterisk) - mitochondria; MF - myelin fibers; N - nucleus; NM - nuclear membrane; d.p.i. - days post infection.

**Figure 6:**
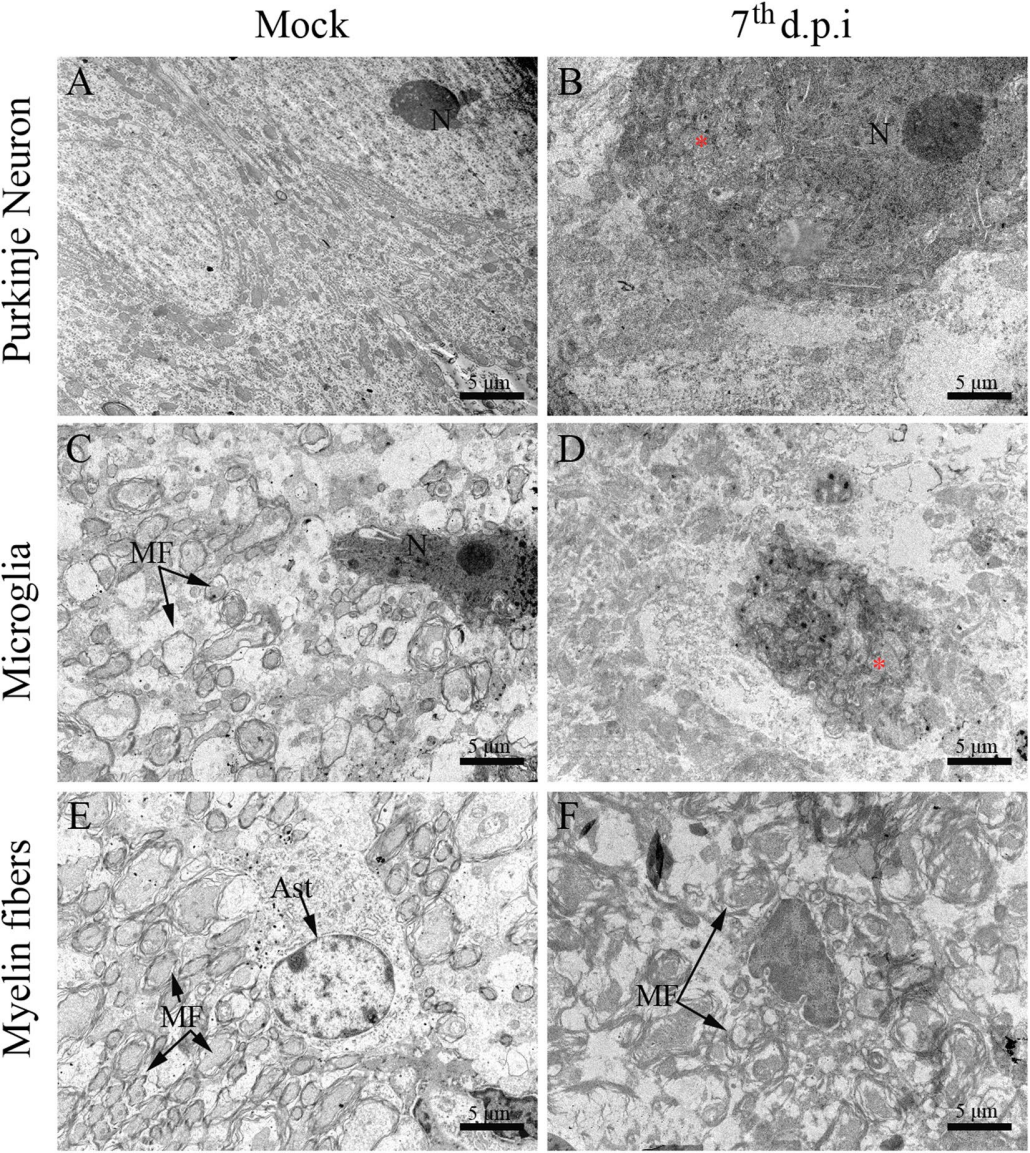
Ultrastructural aspects of the cerebellum tissue of BALB/c mice infected with DENV-2. (**A)** Pyramidal neurons, **(C)** microglial cells and **(D)** organized myelin fibers in samples from non-infected mice. Samples from infected mice showing **(B)** Purkinje neuron with increased nucleus and swollen mitochondria, **(D)** microglial cell with swollen mitochondria and **(F)** disorganized myelin fibers. Samples from infected mice were considered on the 7^th^ d.p.i. M - mitochondria; MF - myelin fibers; N - nucleus; d.p.i. - days post infection.

To obtain a better description of the cellular changes in the CNS originated upon infection with DENV, ultrastructural evaluations were performed using electron microscopy. Brain and cerebellum tissues from infected mice showed degenerated pyramidal and Purkinje neurons with irregular nuclear membrane and mitochondria swelling (Figs 5B and 6B). In the same analyzed sites, microglial cells exhibited increased nucleus and loss of integrity of mitochondrial ridges (Figs 5D and 6D). Brain astrocytes showed a deposition of heterochromatin in a cell pole (Fig. 5F). In the cerebellum, there was disorganization of the myelin fiber pattern suggesting a process of demyelinating neuropathy (Fig. 6F), which corroborated with the findings from the histopathological analyses. Samples collected from control mice exhibited regular structures of pyramidal and Purkinje neurons (Fig. 5A and 6A), microglial cells (Fig. 5C and 6C), astrocytes (Fig. 5E) and myelin fibers (Fig. 6E).

### Viral detection in the SNC of DENV-infected mice

**Figure 7:**
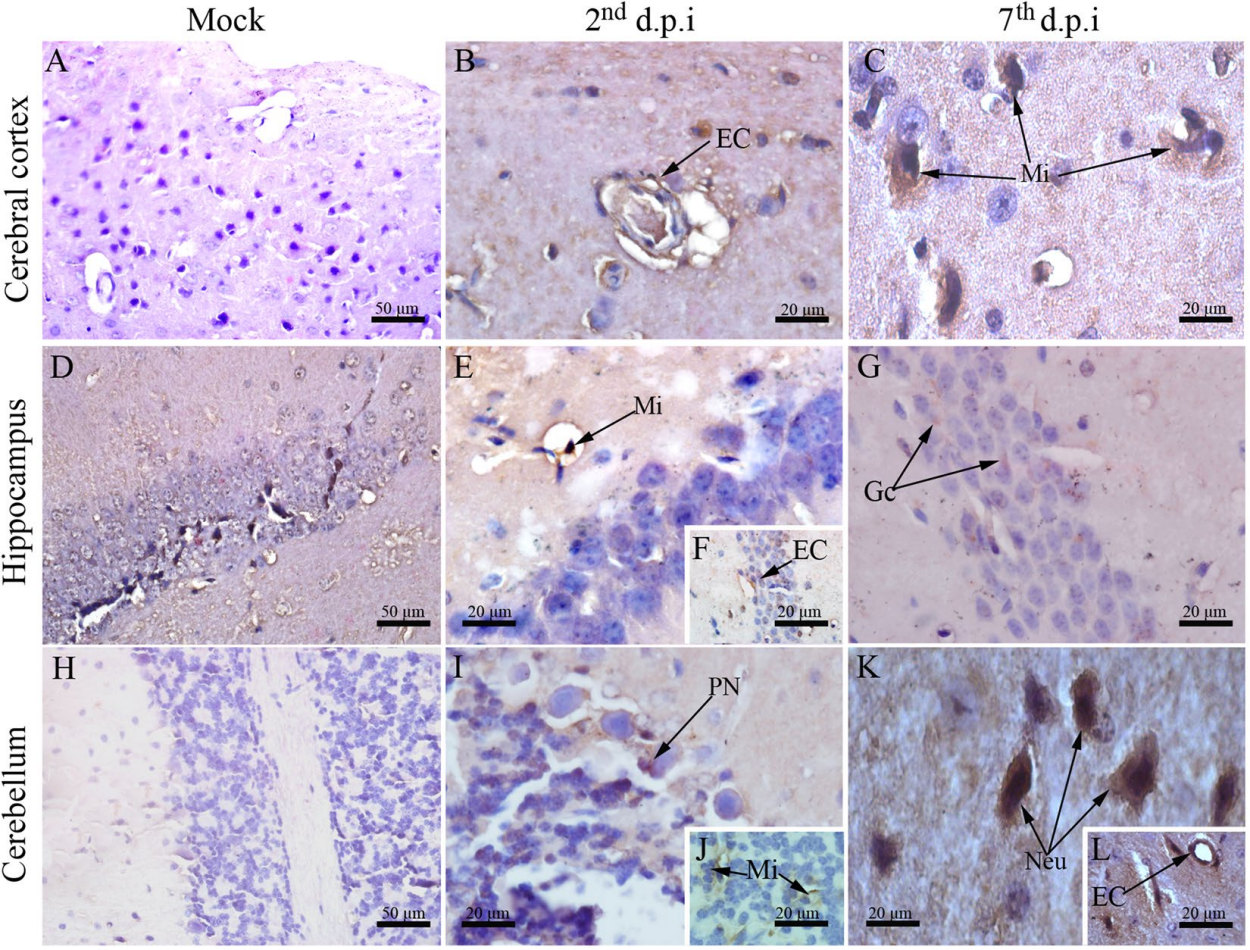
Detection of DENV-NS3 protein in brain and cerebellum tissues. DENV-NS3 protein was detected using immunohistochemistry on brain and cerebellum cuts. Regions of **(A)** cerebral cortex, **(D)** hippocampus and **(H)** cerebellum from samples of control mice showing negative staining reaction for DENV-NS3. Samples from infected animals showing detection of DENV-NS3 in: **(B)** endothelial and **(C)** microglial cells located at the cerebral cortex on the 2^nd^ and 7^th^ d.p.i., respectively; **(E**,**F)** microglial/endothelial cells and **(G)** granular cells of the hippocampus on the 2^nd^ and 7^th^ d.p.i., respectively; **(I**,**J)** Purkinje neurons/microglial cells and **(K**,**L)** neurons/endothelial cells on the 2^nd^ and 7^th^ d.p.i., respectively. EC - endothelial cell; Mi - microglial cells; Gc - granular cells; PN - Purkinje neurons; Neu - neurons; d.p.i. - days post infection.

To address the viral presence and its ongoing replicative process in the CNS we performed immunostaining of DENV-NS3 protein, since this viral antigen is only expressed upon viral replication. DENV-NS3 protein was found in endothelial cells of the cerebral cortex and hippocampus (Fig. 7B,F), microglial cells of the hippocampus and cerebellum (Fig. 7E,J) and in Purkinje neurons on the 2^nd^ d.p.i. (Fig. 7I). On the 7^th^ d.p.i., DENV-NS3 was present in microglial cells of the cerebral cortex (Fig. 7C), granular cells of the hippocampus (Fig. 7G), neurons and endothelial cells in the cerebellum (Fig. 7K,L).

### Measurement of viral titers in the infected brains

Brain cuts from infected mice showed clear expression of DENV-NS3 in several cell types. In order to strengthen this observation a new round of experiment was performed in which brains from infected animals were collected on the 2^nd^ (10 mice) and on the 7^th^ d.p.i. (9 mice). Mock-infected controls (4 animals per group) were also taken into account on the 2^nd^ and on the 7^th^ day after inoculation. While all controls were negative, four animals out of ten had their brain tissue positive for DENV-RNA on the 2^nd^ d.p.i. with titers of 3.72 × 10^4^, 5.68 × 10^4^, 6.02 × 10^3^ or 9.19 × 10^2^ RNA copies/mL. Two animals out of nine were positive for DENV-RNA in their brains on the 7^th^ d.p.i. with titers of 3.80 × 10^4^ or 2.26 × 10^3^ RNA copies/mL.

## Discussion

In this work, using BALB/c animals inoculated with DENV-2 strain 66985 by the i.v. route, we characterized several alterations in the CNS of infected mice which could be correlated with the neurological component of the disease. We identified that brain and cerebellum tissues extracted from DENV-infected animals presented cellular damage, tissue disorganization, morphological alterations of microglia and astrocytes (cells that provide support and homeostatic balance to neurons) and ultrastructural cellular impairments that suggested apoptotic induction. We also found that DENV was able to reach the brain and cerebellum tissues of mice upon i.v. inoculation, an observation that supported the natural neurotropic behavior of the considered viral strain.

Although the infected animals presented these CNS alterations, an intriguing observation that must be taken into account is that the animals were not phenotypically influenced after virus inoculation. Survival rates were not affected by the infection. Additionally, previous observations showed that animals remained apparently asymptomatic for at least 50 days after viral inoculation^26^. We consider that the absence of clinical effects in infected mice, such as altered motor coordination for example, did not exclude the possibility of other existing effects affecting the CNS. A study that corroborates with this line of thinking is from Huy and colleagues that by means of a meta-analytical study identified that clinical manifestations, such as, restlessness, irritability, dizziness, drowsiness and stupor have been associated to the disease^24^. Given the empirical nature in assessing these clinical parameters in animal models, it becomes difficult to categorically affirm whether infected mice were experiencing neurological symptoms at this peculiar level or not. Another fact that may have contributed to the absence of apparent CNS-related effects in infected mice is that immunocompetent animals are naturally resistant to DENV infection. It was seen that DENV is not able to subvert the IFN-*α*/*β* antiviral response in mice^31–33^ as it happens in humans^34–42^. Despite this resistance, immunocompetent mice can still be infected as evidenced in other mouse models of dengue^26,43–46^. However, these animal models do not reproduce the full specter of symptoms as characterized in humans. In our experimental approach, even considering this phenotypic peculiarity, we could observe signs of host response to the infection, such as APC induction and TCD8 cell activation. Additionally, by an unrecognized mechanism, viruses were able to cause blood-brain barrier dysfunction and infect brain and cerebellum cells. One hypothesis to explain this scenario is that the immune-privileged characteristic of the brain may be related to increased permissiveness of viral replication in this site. Our data also showed that the infection leads to circulatory disturbances (hemorrhage), which on its own infers changes in the permeability of the blood-brain barrier. Although hemorrhagic events were scarce, they would have been sufficient to cause an early outbreak of the virus (on the 2^nd^ d.p.i.) from the circulation to the brain.

Among the tissue alterations found in the infected mice, we observed inflammatory infiltrates, thickening of pia matter, increased number of activated neuroglial cells and DENV-NS3 expression in brain and cerebellum cells. Such alterations were in accordance with the study from Amaral and coworkers, in which after infecting C57BL/6 mice intracerebrally with DENV-3, the following changes were observed: (i) increased leukocyte rolling and adhesion in brain microvasculature; (ii) tissue evidences of meningoencephalitis, such as perivascular hemorrhages and infiltration of mononuclear cells in brain and cerebellum; (iii) reactive gliosis; and (iv) immunoreactive cells for anti-NS3 in several brain areas^47^. While these observations were essentially in congruence to our study, the phenotype of infected C57BL/6 mice diverged from our infected mice. In Amaral and coworkers’ model, animals showed symptoms of apathy, stereotyped behavior, seizures and died at the 8^th^ d.p.i., suggesting the induction of an intense encephalitis by the viral infection. The effectiveness of this referred model in promoting such a drastic neurological manifestation may be involved with the following reasons: (i) infection was administered by the intracranial route that obviously resulted in a massive viral load in the host’s CNS; (ii) animals were infected with DENV-3 genotype I and its level of virulence in comparison to the strain we used is unknown; and (iii) divergences of mouse strain susceptibility to DENV (C57BL/6 versus BALB/c) is also unknown. Based on the above reasons, we consider that the histological alterations found in our infected mice resembles the scenario of encephalitis, however in a much more limited fashion that is not able to yield detectable motor alterations or death. Yet, this controlled and relatively slow process by which the virus invades the CNS, spread and promote the specific changes found in our experimental approach can provide us hints about how the neurological manifestations of dengue take place.

Infected animals responded to virus with induction of TCD8-mediated immunity. However, this host response was still unable to clear the virus, as within two d.p.i. the pathogen could able to reach the brain and to promote alteration in the tissue organization. In the normal pathway of infection, infected mosquitoes deliver the virus to the subcutaneous space. What is generally expected when the infection bypasses the innate immunity, is an initial activation of APCs, migration of these activated cells to lymphoid organs and further expansion and distribution of the virus to the circulation. In our approach, the virus could have reached the brain relatively fast (on the 2^nd^ d.p.i.) because it was administered directly by the i.v. route. Also, if we look at the TCD8 activation kinetic, a relevant specific clone expansion was not seen at the 2^nd^ d.p.i., instead, it was detected by the 7^th^ d.p.i. Consistent to this, major perivascular and pia mater infiltrates were detected by the 7^th^ d.p.i. and not by the 2^nd^ d.p.i. Taking together, in this peculiar case, the virus may have took advantage during the initial days of infection to reach and infect the brain while the TCD8 response was still starting to develop.

Neurogliosis found in the brain of infected animals may represent a key finding in dengue neuropathogenesis. Due to morphological changes and increased cell numbers, it became clear that microglial cells (IBA-1^+^) and astrocytes (GFAP^+^) were involved in activation processes. Microglial cells are the resident macrophages of the brain and are known to be susceptible to flavivirus infection^48^. Once activated, microglial cells are thought to act as the first line of defense in the brain tissue as these cells promote antiviral responses to prevent the progression of encephalitis^49^. In their non-activated physiological state, microglial cells display a ramified surface that is suitable for the constant surveillance of the local environment^50^. On the other hand, in pathological conditions these cells become activated and proliferate, assuming an ameboid form that is usually characterized as a spherical-shaped cell that carries several phagocytic vacuoles into its cytoplasm^51^. Activated microglia release many factors that contribute to inflammation and tissue repair^52,53^, however, exacerbated reactions of these cells are implicated with massive production and release of IFN-*γ*, TNF-*α* and nitric oxide, which can be toxic to neurons. In fact, it represents a mechanistic arm that influences several CNS pathologies, such as multiple sclerosis^54,55^, Alzheimer’s disease^56^ and Parkinson’s disease^57^. In our experimental approach, it is also likely that microglial cells can function as a “double-edged sword” either by acting protectively when promoting the initial antiviral response, or by mediating local damage or dysfunction in cases of signaling exacerbation. Reactive gliosis was also characterized in our experimental approach of infection by the presence of clusters of astrocytes (GFAP^+^ cells). Astrocytes are the most abundant cells in the CNS and act cooperatively with microglia to stimulate T-cell responses^58^. Divergences in morphology, when we compared infected to non-infected samples, also suggested activation of this cell subpopulation upon DENV infection. However, in spite of limitations considering the used cell marker^59^ and the controversial behavior typical of astrocytes in promoting/inhibiting inflammation^60^, more investigation is needed to better characterize the contribution of these cells in the considered scenario.

Other findings that were implicated with the establishment of neuropathological processes were resultant from the ultrastructural analyses. We observed that neurons, microglia and astrocytes in the brain/cerebellum areas of infected mice exhibited cellular alterations (mainly mitochondria swelling) that indicated processes of apoptotic cell death. The induction of apoptosis by DENV in susceptible cells is well reported in the literature^61–64^. Particularly, mitochondria swelling in response to DENV was previously characterized under other circumstances such as in peripheral organs of dengue fatal cases^65^ and in human hepatoma cell line (Hep-G2)^66^. Similarly to other viral diseases, DENV proteins could also interact with mitochondrias and somehow result in the modulation of apoptotic processes^67,68^. Given the central role of the brain in receiving information from the body, interpreting and then guiding the body’s response to it, the elimination of brain cells by apoptotic processes could be critical depending on the affected areas. As these target cells, neurons and neuroglial cells, are located within the cerebral cortex and white matter, respectively, it is reasonable to suggest that DENV infection could potentially impact the host’s sensory and/or motor functions. An additional effect that could also potentially commit the motor functions is the demyelination found in the cerebellar tissue. Previous studies suggested that there is a connection between the disruption of the myelin sheath and the activation of microglia^69^, nonetheless, in dengue this phenomenon is still unknown.

In conclusion, we observed that DENV-2 strain 66985 isolated from the patient and subsequently administered by the i.v. route in mice was able to cause dysfunction in the blood-brain barrier and to replicate in endothelial cells of the brain. This occurred in association with tissue damage/disorganization of CNS structures, reactive gliosis and induction of apoptotic cell death. It is important to note that not all dengue infections lead to neurological manifestations, hence, the phenomena observed in this work could be influenced by the viral strain or even by the applied dose. The experimental approach of infection presented in this work reaffirmed the neurotropic nature of the considered DENV strain in mice and shed light into possible existing CNS changes that may justify neurological manifestations in dengue disease.

## Methods

### Virus

The virus used in our experiments was the DENV-2 strain BR/RJ66985/2000 representing the linage I isolated from human serum samples at Laboratório de FlavivÃrus/Instituto Oswaldo Cruz (IOC)/Fiocruz/Rio de janeiro/Brazil. The BR/RJ66985/2000 strain (GenBank register number: #HQ012518) was isolated in 05/05/2000 from a 39-year-old male patient that presented classic dengue^70^. Viruses were propagated in *Aedes albopictus* mosquito cell line (C6/36) using L-15 medium (Sigma, USA) supplemented with 1% non-essential amino acids, 10% tryptose phosphate broth solution and 10% fetal bovine serum. Infected cells were cultured at 28 °C for 15 days. Viruses were isolated and identified by indirect immunofluorescence technique using 3H5 monoclonal antibody which is type-specific for DENV-2^71^. Finally, the progeny viruses were titrated in C6/36 cells according to Reed and Muench method^72^.

### Ethics in experimental procedures and mice infection

All experiments with mice were conducted in compliance with Ethical Principles in Animal Experimentation stated in the Brazilian College of Animal Experimentation and approved by the Institute’s Animal Use Ethical Committee (acceptance protocol P-12/11-3). Mice were grouped with n = 12.

Adult male BALB/c mice, 2 months old, received 20 *μ*l of DENV-2 at a concentration of 100 TCID_50_ intravenously by the caudal vein. On the 2^nd^ or 7^th^ day post infection (d.p.i.), animals were anesthetized with a mixture of ketamine-xylazine^73^, sacrificed and then brain and cerebellum tissues were collected for further analysis (optical and electron microscopy). Brains and cerebellums used as controls (mock) were collected from mice that received cell culture (C6/36) supernatants only by the same route and sacrificed at the 7^th^ d.p.i.

### Flow cytometry

For flow cytometry analysis, leukocytes were isolated from blood and spleen. Spleens were dissociated in wire mesh screens using RPMI medium. Spleen macerates and total blood samples were treated with BD FACS Lysing for red blood cell lysis and fixation according to manufacturer’s instructions (BD Biosciences, USA). Cells were spinned down, washed and suspended in PBS/BSA 1%. Approximately 10^6^ cells were stained on ice for 20 min in the dark with the following mab combinations: (i) CD11c-PE and CD86-FITC; or (ii) CD4-PE, CD8-PerCP and CD45RB-FITC. All mabs used in this work were obtained from BD Biosciences and background-staining controls were performed using isotypes recommended by the manufacturer. Samples were read in a BD Accuri C6 flow cytometer and analyzed offline with C6 software (BD Biosciences).

### Histopathological analysis

#### Staining protocol

Histological analyses were carried out based as previously described by Paes and colleagues^26^. Briefly, fragments of brain and cerebellum collected from mice were fixed in 10% buffered formalin, cleaved into smaller fragments, dehydrated in ethanol, clarified in xylene and blocked in paraffin resin. In sequence, samples were sectioned in 5-*μ*m thick units, deparaffinized in xylene and rehydrated with alcohol. Samples were stained with hematoxylin and eosin (H.E.) and visualized under a light microscopy (Olympus BX 53 F, Japan). Digital images were rendered using Image Pro Plus software version 4.5.

#### Quantification of damage

Three types of damage were considered for quantification: hemorrhage, perivascular infiltrate and pia mater infiltrate. Histological brain cuts of animals belonging to each analyzed group (mock, 2^nd^ d.p.i. or 7^th^ d.p.i. -4 animals per group) had 30 images randomly captured at 400× magnification using Image Pro software version 4.5. Tissue damages were quantified and qualified in each of the 30 images as: 0 - absent; 1 - light and focal; 2 - light; 3 - moderate; and 4 - diffuse. Infected animals considered in this analysis were positive for DENV-NS3 immunohistochemical reaction in the brain (see “Immunohistochemistry” section in “Methods”).

### Ultrastructural analysis

For electron microscopy, fragments of brain and cerebellum were post fixed with 2.5% glutaraldehyde in sodium cacodylate buffer (0.2 M, pH 7.2), dehydrated in acetone, post fixed with 1% buffered osmium tetroxide, embedded in EPON and polymerized at 60 °C for three days. Semi-thin sections (0.5 *μ*m thick) were obtained using a diamond knife (Diatome, Switzerland) adapted to a Reichert-Jung Ultracut E microtome (Markham, Canada) and stained with methylene blue. Ultra-thin sections (60–90 nm) were contrasted with uranyl acetate^74^ and lead citrate^75^ and observed under a JEOL-JEM-1011 transmission electron microscope.

### Immunohistochemistry

#### Staining protocol

For immunohistochemical studies, the paraffin-embedded tissues were cut (5 *μ*m thick), incubated at 60 °C for one hour, deparaffinized in xylene and rehydrated with alcohol. Antigen retrieval was performed by heating the tissue in the presence of citrate buffer. Next, tissues were blocked for endogenous peroxidase with 3% hydrogen peroxidase in methanol for 10 minutes and rinsed in tris-HCl (pH 7.4). To reduce non-specific binding, sections were incubated in Protein Blocker solution (Spring Bioscience, USA) for 10 min at room temperature. Afterwards, samples were incubated overnight at 4 °C with primary antibodies: (i) anti-IBA1 antibody (1:200, Wako) to recognize microglia cells; or (ii) anti-GFAP antibody (1:300, Sigma) for astrocyte staining; or (iii) anti-NS3 antibody (diluted 1:100, to detect the DENV-infected cells. In the next day, sections were incubated with secondary complement (REVEAL complement - Spring Bioscience) for 10 minutes and with a rabbit anti-mouse IgG-HRP conjugate (REVEAL polyvalent HRP - Spring Bioscience) for 15 minutes at room temperature. Reactions were revealed with diaminobenzidine (Spring Biosciense) as a chromogen and then sections were counterstained with Meyer’s hematoxylin (Dako). Finally, samples were analyzed under an Olympus BX 53 microscope and frames were acquired using a coupled Olympus DP72 camera.

#### Quantification of positive cells

For each specific staining (IBA-1 and GFAP) 50 images were randomly captured at 1000× magnification using Image Pro software version 4.5. Positive cells were quantified in each of the 50 images and the median of positive cell number was determined. All analyzes were accomplished in a blind test without prior knowledge of the studied groups. After quantification, frames exhibited in figures were selected as to be more informative according to specific areas in the analyzed tissues.

### Immunofluorescence assay

The paraffin-embedded tissues were cut (5 *μ*m thick), incubated at 60 °C for one hour, deparaffinized in xylene and rehydrated with alcohol. Antigen retrieval was performed by heating the tissue in the presence of citrate buffer. The sections were permeabilized for 10 minutes with 0.5% Triton X-100, and incubated for 30 minutes with 2% bovine serum albumin (BSA) and 5% normal goat serum (NGS) for blocking, at room temperature. In sequence, slides were co-stained overnight at 4 °C with anti-F4/80 (eBioscience) and anti-NS3^65^ (in-house produced antibody) antibodies diluted at 1:200 and 1:100, respectively. Sections were washed in PBS and incubated with Alexa 488-conjugated rabbit anti-mouse IgG (Thermo Scientific) or with Alexa 555-conjugated goat anti-rabbit IgG (Thermo Scientific, USA). Samples were analyzed under a Zeiss LSM 510 Meta confocal microscope (Zeiss, Germany).

### Quantitative polimerase chain reaction (qPCR)

DENV viral titers in the brain of mice were estimated by using the quantitative qPCR system Taqman (PE Applied Biosystems, Foster City, CA, USA) according to the protocol described by Johnson and coworkers^76^. For the analysis by molecular techniques, the viral RNA was extracted from samples using the QIAamp Viral RNA Mini Kit (Qiagen, Hilden, Germany) following the manufacturer’s instructions. The estimated limit of detection for the technique was 1.0 × 10^−5^ DENV2-RNA copies per mL.

### Statistical Analysis

Data obtained from the quantification of positive cells in the immunohistochemistry were analyzed with GraphPad prism software v5.1 (La Jolla, USA) using non-parametric statistical tests. Significant differences between analyzed groups (mock, 2^nd^ d.p.i. and 7^th^ d.p.i.) were determined using Mann-Whitney test with *p < 0.05.

### Data Availability

All data generated or analyzed during this study are included in this published article.

## Electronic supplementary material

Supplementary Table1
