# Supplementary material for: BALB/c mice infected with DENV-2 strain 66985 by the intravenous route display injury in the central nervous system
Source: Sci Rep. 2018 Jun 27;8:9754. doi: 10.1038/s41598-018-28137-y (PMC6021404; doi:10.1038/s41598-018-28137-y)
Supplement: Supplementary file 1 — Supplementary Table1 [file 41598_2018_28137_MOESM1_ESM.pdf]

BALB/c mice infected with DENV-2 strain 66985 by the intravenous route display injury in the central nervous system

Natália G. Salomão, Kíssila Rabelo, Tiago F. Póvoa, Ada M. B. Alves, Simone M. da Costa, Antônio J. S. Gonçalves, Juliana F, Amorim, Adriana S. Azevedo, Priscilla C. G. Nunes, Carlos A. Basílio-de-Oliveira, Rodrigo P. Basílio-de-Oliveira, Luiz H. M. Geraldo, Celina G. Fonseca, Flávia R. S. Lima, Ronaldo Mohana-Borges, Emiliana M. Silva, Flávia B. dos Santos, Edson R. A. Oliveira and Marciano V. Paes

Table 1 – Quantitation of damage

0 – absent; 1 – light and focal; 2 – light; 3 – moderate; and 4 – diffuse

Mock

Hemorrhage				perivascular infltrate				Pia mater infltrate			
animal1	animal2	animal3	animal4	animal1	animal2	animal3	animal4	animal1	animal2	animal3	animal4
0	0	0	0	0	0	0	0	0	0	0	0
0	0	0	0	0	0	0	0	0	0	0	0
0	0	0	0	0	0	0	0	0	0	0	0
0	0	0	0	0	0	0	0	0	0	0	0
0	0	0	0	0	0	0	0	0	0	0	0
0	0	0	0	0	0	0	0	0	0	0	0
0	0	0	0	0	0	0	0	0	0	0	0
0	0	0	0	0	0	0	0	0	0	0	0
0	0	0	0	0	0	0	0	0	0	0	0
0	0	0	0	0	0	0	0	0	0	0	0
0	0	0	0	0	0	0	0	0	0	0	0
0	0	0	0	0	0	0	0	0	0	0	0
0	0	0	0	0	0	0	0	0	0	0	0
0	0	0	0	0	0	0	0	0	0	0	0
0	0	0	0	0	0	0	0	0	0	0	0
0	0	0	0	0	0	0	0	0	0	0	0
0	0	0	0	0	0	0	0	0	0	0	0
0	0	0	0	0	0	0	0	0	0	0	0
0	0	0	0	0	0	0	0	0	0	0	0
0	0	0	0	0	0	0	0	0	0	0	0
0	0	0	0	0	0	0	0	0	0	0	0
0	0	0	0	0	0	0	0	0	0	0	0
0	0	0	0	0	0	0	0	0	0	0	0
0	0	0	0	0	0	0	0	0	0	0	0
0	0	0	0	0	0	0	0	0	0	0	0
0	0	0	0	0	0	0	0	0	0	0	0
0	0	0	0	0	0	0	0	0	0	0	0
0	0	0	0	0	0	0	0	0	0	0	0
0	0	0	0	0	0	0	0	0	0	0	0
0	0	0	0	0	0	0	0	0	0	0	0

2nd d.p.i.

Hemorrhage				Perivascular infiltrate				Pia mater infiltrate			
animal1	animal2	animal3	animal4	animal1	animal2	animal3	animal4	animal1	animal2	animal3	animal4
0	0	0	0	0	1	3	0	0	0	0	0
0	0	0	0	0	0	0	0	0	0	0	0
0	0	0	0	0	0	0	0	0	0	0	0
0	0	0	0	1	1	0	1	0	0	0	0
0	0	0	0	0	0	0	0	1	0	0	2
0	0	0	0	1	0	1	0	0	0	0	0
0	0	0	0	1	0	0	0	0	0	0	0
0	0	0	0	0	0	0	0	1	1	2	0
0	0	0	0	0	0	0	0	0	0	0	0
0	0	0	1	0	2	0	0	0	0	0	0
0	0	0	0	0	0	0	0	0	0	0	1
0	0	0	0	0	2	0	0	0	0	0	0
1	0	0	0	0	0	4	0	0	0	0	0
0	0	0	2	0	0	3	2	0	0	0	1
0	0	0	0	0	0	3	0	1	0	0	0
0	0	0	0	0	0	3	0	0	0	0	0
0	1	0	0	0	0	2	1	0	0	0	0
0	0	0	0	0	0	0	1	0	0	0	0
0	0	0	0	0	0	0	0	0	0	0	1
0	0	0	0	0	0	0	0	1	0	0	0
0	0	0	1	0	0	0	0	1	0	0	0
0	0	0	0	0	0	0	0	0	0	0	0
0	0	0	0	0	0	0	0	0	0	0	0
0	1	0	0	0	0	0	1	0	0	1	0
0	1	0	0	1	0	0	0	0	0	0	0
0	0	0	0	0	0	0	0	0	0	0	0
0	0	0	0	0	0	0	1	2	0	0	0
0	0	0	0	0	0	0	0	0	0	0	0
0	0	0	0	0	0	1	0	0	0	0	0
0	0	0	0	0	1	1	0	0	0	0	0

7th d.p.i.

Hemorrhage				Perivascular infiltrate				Pia mater infiltrate			
animal1	animal2	animal3	animal4	animal1	animal2	animal3	animal4	animal1	animal2	animal3	animal4
0	0	0	0	1	0	1	0	1	1	1	0
0	0	0	0	0	0	0	0	1	0	0	2
0	0	3	0	0	0	2	1	0	0	0	0
0	3	0	0	1	0	0	4	0	1	0	0
0	0	0	0	0	0	0	0	0	2	0	1
0	0	0	0	0	2	0	0	0	0	0	0
0	0	1	0	0	1	1	0	0	0	0	0
1	0	2	0	0	0	0	0	1	0	2	0
0	2	0	0	0	0	3	3	0	0	0	0
0	0	0	0	0	0	0	0	0	0	0	0
0	0	0	1	0	0	3	0	0	0	0	1
0	0	0	0	4	0	0	0	0	0	1	1
0	0	0	0	0	1	0	0	0	0	0	1
0	0	0	0	0	1	4	0	0	0	1	0
0	0	0	1	1	0	0	0	0	0	0	0
0	0	1	0	0	3	0	2	1	0	0	2
0	0	0	0	0	2	0	0	0	1	1	1
0	0	0	0	2	1	2	3	0	0	0	0
0	0	0	0	0	0	0	3	0	0	0	0
0	0	0	0	0	0	0	1	0	0	0	2
0	0	0	0	0	0	0	0	2	0	0	0
0	0	1	0	0	0	1	2	0	0	0	0
0	2	0	0	1	0	0	0	0	0	0	0
0	0	2	0	1	1	0	2	0	0	0	0
2	0	0	0	1	0	2	0	2	0	0	0
0	0	0	0	0	0	2	1	0	2	0	0
0	0	1	0	0	1	1	2	0	0	0	0
0	0	0	0	3	0	0	0	0	1	1	0
0	0	0	1	0	0	1	0	0	0	0	0
0	0	1	0	3	0	1	0	1	0	0	0
